# This Package Came With a Bite: An Unusual Case of Cutaneous Leishmaniasis

**DOI:** 10.51894/001c.164567

**Published:** 2026-07-31

**Authors:** Ali El Moussa, Karim Khanafer, Mohammad H. Ayoub, Abe Al Hage

**Affiliations:** 1 Henry Ford - Wyandotte

## 61

### Introduction

Cutaneous leishmaniasis is typically associated with travel to endemic regions and may be overlooked in patients without conventional exposure history. In the United States, this can delay diagnosis when lesions mimic more common conditions such as cellulitis.

### Case Presentation

A 36-year-old man in Michigan presented with a progressive right lower extremity lesion that worsened despite multiple courses of empiric antibiotics prescribed for presumed cellulitis. He reported that the lesion began after an insect bite sustained immediately after opening a package of organic plants shipped from Florida to Michigan. He later clarified that he had not traveled to Florida and that the suspected exposure occurred locally in Michigan. The lesion enlarged over several months with worsening discoloration and pruritus, without purulent drainage or significant systemic symptoms.

### Methods

Clinical history, exposure history, emergency department documentation, laboratory and imaging findings, dermatologic evaluation, and skin biopsy results were reviewed.

### Results

Laboratory evaluation, including CBC, BMP, ESR, and CRP, was not suggestive of severe systemic bacterial infection. Imaging of the right tibia and fibula showed no fracture, radiopaque foreign body, or soft tissue gas. The patient was admitted for intravenous antibiotics because of lesion progression and failure of prior outpatient treatment, although suspicion for systemic sepsis remained low. He was discharged with outpatient dermatology follow-up. Subsequent skin biopsy demonstrated organisms morphologically consistent with Leishmania amastigotes, confirming cutaneous leishmaniasis. He was treated with cutaneous cryotherapy and amphotericin B, with gradual improvement over 6 to 7 months.

### Conclusion

This case highlights the importance of reconsidering the diagnosis in chronic or progressive cellulitis-like lesions that fail repeated antibiotic therapy. Early biopsy and broader infectious evaluation should be pursued, even in patients without travel history, as unusual exposure histories may provide critical diagnostic clues.

